# Immunomodulatory function of chitosan is dependent on complement receptor 3

**DOI:** 10.1016/j.tcsw.2025.100146

**Published:** 2025-05-22

**Authors:** Jeanette Wagener, Xiaowen Wang, Katharina L. Becker, Vishu Aimanianda, Isabel Valsecchi, Mark S. Gresnigt, Mihai G. Netea, Jean-Paul Latge, Neil A.R. Gow, Frank L. van de Veerdonk

**Affiliations:** a Medical Research Council Centre for Medical Mycology at the University of Exeter University of Exeter, Geoffrey Pope Building, Stocker Road, Exeter EX4 4QD, UK; b Department of Internal Medicine and Radboudumc Center for Infectious Diseases, Nijmegen, the Netherlands; c Department of Dermatology, Peking University First Hospital, Beijing, China; d Unité des Aspergillus, Institut Pasteur, Paris, *France.*^*5*^Aberdeen Fungal Group, School of Medicine, Medical Sciences & Nutrition, Institute of Medical Sciences, University of Aberdeen, Foresterhill, Aberdeen AB25 2ZD, UK; e Junior Research Group Adaptive Pathogenicity Strategies, Leibniz Institute for Natural Product Research and Infection Biology – Hans Knöll Institute, Jena, Germany; f Department of Immunology and Metabolism, Life and Medical Science Institute, University of Bonn, Germany

**Keywords:** Cell wall, Chitosan, Fungal immunology, Pattern recognition receptor

## Abstract

Chitosan, the deacetylated product of chitin, is a significant component of the cell walls of nearly all fungi. In contrast with the high level of attention paid to plant immune recognition of chitin and chitosan of plant pathogenic fungi we know much less about the mammalian immune system immune recognition of chitosan during infections by human pathogenic fungal species. Here we show that the mammalian β-integrin CR3 complement scavenger receptor, that is expressed on monocytes and macrophages, recognises chitosan from a range of fungal sources and that this leads to the secretion of IL-6, IL-1β and TNF. The secretion of these pro-inflammatory cytokines was dependent on the phagocytosis of chitosan. The co-provision of chitosan along with a peptide (Aspf2 from *Aspergillus fumigatus*) presented by the MHCII complex potentiated a Th response leading to IL-22 production. Fungal cell wall chitosan therefore activates both the innate and adaptive arms of the human immune system.

## 1 Introduction

Chitin, a β-(1,4)-linked polymer of *N*-acetylglucosamine (GlcNAc), is the second most common polysaccharide in nature, and one of the key components of fungal cell walls. Chitosan, a β-(1,4)-linked polymer of glucosamine (GlcN), the deacetylated derivative of chitin, is less prevalent as chitin in nature, but it is present in medically important fungi, namely *Aspergillus fumigatus*, *Cryptococcus neoformans* (Baker et al., 2011; Bueter et al., 2013; Sanchez-Vallet et al., 2015; Lam et al., 2019; Shih et al., 2019), and in members of the mucoraceous fungi such as *Mucor* spp. and Rhizopus spp. (Geoghegan and Gurr, 2016; Ye et al., 2020; Guo and Cheng, 2022; Bakhat et al., 2023) (Cheng et al., 2024). Fungal chitosan has been shown to be important for fungal pathogenesis for both plant (Chandra et al., 2015, Sanchez-Vallet et al., 2015, Geoghegan and Gurr, 2016, Ye et al., 2020, Guo and Cheng, 2022, Bakhat, Vielba-Fernandez et al. 2023)) and human pathogenic fungal species (Baker et al., 2011, Bueter et al., 2013, Upadhya et al., 2018, Lam et al., 2019, Shih et al., 2019). Chitosan is a chemically versatile material and it is frequently used in pharmaceutical, cinical and commercial applications (Meng et al., 2021) such as gene and drug delivery constructs (Saravanakumar et al., 2020), tissue scaffolds (Kim et al., 2018), wound dressings (Hurler and Skalko-Basnet, 2012) and as vaccine adjuvants (Li et al., 2013, Komi et al., 2018, Fong and Hoemann, 2018, Lin et al., 2022, Santos Junior et al., 2023).

Humans do not synthesise chitin or chitosan, but they do express chitin and chitosan degrading enzymes such as chitinases, chitosanases and lysozyme (Kumar and Zhang, 2019). Humans additionally express chitinase-like binding proteins (CLPs) (Di Rosa et al., 2016). Differential expression of chitinases and CLPs have been described in various immunological processes and diseases so far, such as asthma or rheumatoid arthritis (Di Rosa, Distefano et al. 2016). The human innate immune system recognises pathogen associated molecular patterns (PAMPs) released by the degradation of bacterial and fungal cell walls through pattern recognition receptors (PRRs) (Netea et al., 2015, Erwig and Gow, 2016, Lionakis et al., 2023). Recent studies propose different PRRs to be involved in the recognition of fungal chitin with varying immune responses reported (Rementeria et al., 1997, Mora-Montes et al., 2011, Bueter et al., 2013, Wagener et al., 2014, Becker et al., 2016).

In *C. neoformans* chitin and chitosan are crucial for pathogenesis and immune recognition and activation (Baker et al., 2011, Wiesner et al., 2015, Upadhya et al., 2018, Lam et al., 2019). Chitosan that is deacetylated from the chitin made by the Chs3 chitin synthase dampens inflammatory immunity and affects pathogenesis in a medium-dependent fashion (Upadhya, Baker et al. 2018, Hole et al., 2020, Upadhya, Lam et al. 2021, Upadhya, Lam et al. 2023). In this pathogenic yeast chitin is also important for the production of the superficial gelatinous capsule which is major virulence factor of this pathogen. In *C. albicans* chitin has been shown to activate anti-inflammatory responses and macrophage function (Wagener et al., 2017, Walker and Munro, 2020) in which the particle size, source and purification method of the chitin particle is important for the immune response (Mora-Montes, Netea et al. 2011, Alvarez, 2014, Wagener et al., 2014, Walker and Munro, 2020). Also dectin-1-β-glucan mediated recognition responses in vivo has been shown to be influenced by the chitin content of the strain of *C albicans* (Marakalala et al., 2013). Chitin exposure is regulated by cell wall stress such as exposure to the β-glucan antifungal caspofungin (Wheeler and Fink, 2006, Mora-Montes, Netea et al. 2011, Wagner et al., 2023). Chitin also can induce trained immunity – the recently discovered memory system of the innate immune response (Rizzetto et al., 2016).

Unlike fungal chitin, there are few studies available in the literature investigating the immunological properties of fungal chitosan or its recognition by PRRs. However, chitosan, enzymatically or chemically derived from shrimp or crab shell chitin, has been shown to stimulate IL-1β release from multiple murine and human cell types (Bueter et al., 2011a, Bueter et al., 2011b, Bueter et al., 2014). Nonetheless, recognition and interaction of fungal chitosan with immune cells, and the exact receptors and pathways involved remain unknown.

In this study, we aimed to explore the immunological properties and PRR pathways of fungal chitosan, either chemically derived from fungal chitin, or directly extracted from the cell wall of chitosan-containing fungi to understand the nature of the immune response to chitosan and chitosan-containing fungi.

## 2 Materials and methods

### 2.1 Ethics statements

All experiments were performed and conducted in accordance to Good Clinical practice and the Declaration of Helsinki, and further approved by the Arnhem-Nijmegen Ethical Committee (nr.2010/104) and the College Ethics Review Board of the University of Aberdeen (CERB/2012/11/676 & CERB/2016/8/1300). Human blood was, either, collected from healthy volunteers by venepuncture after obtaining informed consent or obtained as buffy coats extracted from healthy volunteers from the Sanquin Bloodbank, Nijmegen (The Netherlands).

### 2.2 Peripheral blood mononuclear cell (PBMC) isolation and stimulation

PBMCs were isolated using Ficoll®-Paque Plus (GE Healthcare) density gradient centrifugation as described elsewhere (van de Veerdonk et al., 2009a, van de Veerdonk et al., 2009b). PBMCs were suspended in RPMI 1640 (Dutch modification, Gibco) supplemented with 50 μg/ml gentamicin, 2 mM l-glutamine and 1 mM pyruvate (Gibco). 0.5 × 10^6^ cells/well were plated in a 96-well round bottom plate (Corning). Stimulation experiments were performed with or without the presence of 10 % (*v*/v) human serum as indicated. Where applicable, cells were pre-incubated for 1 h with inhibitors, before stimuli were added. Cells were incubated at 37 °C with 5 % CO_2_ for 24 h or 7 days until cell lysates and supernatants were collected and stored at −20 °C until further analysis.

### 2.3 Purification of chitin and chitosan

Chitin was isolated from the *A. fumigatus* cell wall (*A. fumigatus* CEA17_Δ*akuB*^KU80^ strain mycelia was collected, after 20 h growth at 37 °C in the liquid Sabouraud medium) according to the method described earlier (Becker, Aimanianda et al. 2016). However, the resultant chitin was purified upon recombinant *endo*- β-(1,3)-glucanase treatment. Chitosan was deacetylated from chitin using high temperature and strong alkali. In addition, chitosans were isolated from a variety of fungal strains listed in (Table 1) grown under following conditions and using different extraction protocols: *A. fumigatus* was grown in Sabouraud dextrose broth (1 % (*w*/*v*) peptone, 4 % (w/v) glucose) at 37 °C with shaking at 200 rpm overnight; *Cryptococcus neoformans* was grown in YPD broth (1 % (w/v) yeast extract, 2 % (w/v) peptone, 2 % (w/v) glucose) at 37 °C with shaking at 200 rpm overnight; and *Mucor circinelloides* was grown in Difco™ potato dextrose broth (BD). Chitin was isolated and purified as described previously (Wagener, Malireddi et al. 2014) and chitosan was precipitated from the supernatant of chitin extractions by neutralizing the acidic pH with 10 M NaOH. Chitin and chitosan extractions were washed intensively before analysed for quantity, purity and degree of deacetylation as described elsewhere (Wagener, Malireddi et al. 2014).

### 2.4 PRR ligands, blockers and other stimuli

*Aspergillus fumigatus* conidia were obtained and isolated as described previously (Gresnigt et al., 2013a, Gresnigt et al., 2013b). *Bartonella quintana* LPS was prepared and purified as described elsewhere and used as a TLR4 inhibitor (100 ng/mL) (Popa et al., 2007). All other used chemicals were purchased and used as follows: isotype control mouse IgG1 (10 μg/mL) (eBioscience); anti-TLR2 (10 μg/mL) (eBioscience); Isotype control goat IgG (10 μg/ml), anti-human β_2_-integrin (anti-CR3 [10 μg/ml]), and isotype control mouse IgG2b (10 μg/ml) were obtained from R&D Systems Minneapolis, MN; cytochalasin D (10 μg/mL, dissolved in DMSO) (Sigma-Aldrich).

### 2.5 Expression and purification of an aspergillus protein Aspf2

Recombinant *A. fumigatus* Aspf2 protein was expressed in GS115(his4) *Pichia pastoris* strain (Invitrogen™). For the Aspf2 expression step, the pHIL-S1 vector (Invitrogen™) was used with a AOX1 promoter which allowed Aspf2 gene to be induced with methanol, and PHO1 signal peptide which enable Aspf2 protein to be secreted into the culture medium. In the multicloning site of pHIL-S1 vector only the Aspf2 cDNA from +61 to +912 bp was cloned, because the Aspf2 signal peptide was removed and replaced with the PHO1 signal peptide and the Aspf2 stop codon was replaced by 6 histidine codons (His-tag). The primers used for Aspf2 amplification from Af cDNA were F: 5’TTCGCTCGAGCCCTCCCTACCTCCCCCGTCCCCATC3’ and R: 5’TGGATCCGTGGTGGTGGTGGTGGTGCGAGATCCGGACTGT3’, containing the.

restriction enzymes *Xho*I and *Bam*HI used to clone Aspf2 into the pHIL-S1 vector. The resulting pHIL-S1-Aspf2 vector was first transformed for amplification into *E. coli* on LB-agar with ampicillin, then Sanger sequenced and linearized with *PmeI* to finally be transformed into *P. pastoris* strain GS115(his4).

Aspf2 expression was carried out according to manufacturer's instructions (Pichia expression kit, catalog No. K1710–01, Invitrogen™) where 0.5 %*V*/V of pure methanol was added to the culture medium for recombinant protein induction and added again on the second day of expression to maintain the induction.

For the purification step, the entire culture medium containing recombinant His-tagged Aspf2 protein was first loaded onto the Talon column (Clontech), which was then washed and eluted following the manufacturer's instructions.

### 2.6 Cytokine measurements

IL-1β, TNFα, IL-6, IL-17, IL-22 and IFNγ were measured in the cell culture supernatants using commercial ELISA kits (IL-1β, TNFα, IL-17 and IL-22: R&D Systems; IL-6 and IFNγ: Sanquin) according to the instructions supplied by the manufacturer.

### 2.7 qPCR

RNA was isolated from 1 × 10^6^ PBMCs after stimulation for 24 h using Trizol Reagent (Invitrogen) according to a protocol supplied by the manufacturer. RNA (500 ng) was reverse transcribed into cDNA using the iScript cDNA synthesis kit (Hercules, Bio-Rad Laboratories, CA). Quantitative PCR (qPCR) analysis was performed using SYBR Green Master Mix (Applied Biosystems, Carlsbad, CA) and the Applied Biosystems 7300 real-time PCR system. As PCR protocol the following conditions were used: 2 min 50 °C, 10 min 95 °C followed by 40 cycles at 95 °C for 15 s and 60 °C for 1 min. To correct for differences in loading concentrations of RNA between the different conditions, qPCR results were corrected with the housekeeping gene β2 microglobulin (β2m) amplified using the primers 5′- ATGAGTATGCCTGCCGTGTG-3′ and 5’-CCAAATGCGGCATCTTCAAAC-3′. Primer efficacy was evaluated using a standard curve. Values were compared with the β2m Ct by calculating the delta Ct and the fold-change was calculated relative to the RPMI-stimulated to determine the pro-inflammatory cytokine expression.

### 2.8 Immunoblots

Protein expression was analysed as described before (Wagener, MacCallum et al. 2017). Pro- and mature IL-1β was detected using a monoclonal rabbit anti-IL-1β antibody (clone D3U3E, 1/1000, Cell Signaling), followed by goat anti-rabbit IgG-HRP (1/1000, Cell Signaling); and β-actin was detected using a HRP-conjugated rabbit monoclonal anti- β-actin antibody (clone 13E5, 1/1000, Cell Signaling). Signals were quantified using a Fusion FX7/SL Chemiluminescence and Fluorescence Combination Imaging System.

### 2.9 Statistical analyses

The 2way ANOVA test was used to determine differences between stimulation with and without inhibitors of PRRs, cytokines or cytokine inhibitors. A *P*-value of <0,05 was considered statistically significant and indicated with 1 to 3 stars (*P < 0,05, **P < 0,01 and ***P 〈0,001). Graphs represent cumulative results of all performed experiments and are presented as mean ± standard error of the mean (SEM). Data were analysed with and graphs created with GraphPad Prism.

## 3 Results

### 3.1 Chitosan-induced pro-inflammatory cytokine production by human PBMCs correlates with the degree of deacetylation

**Fig. 1:**
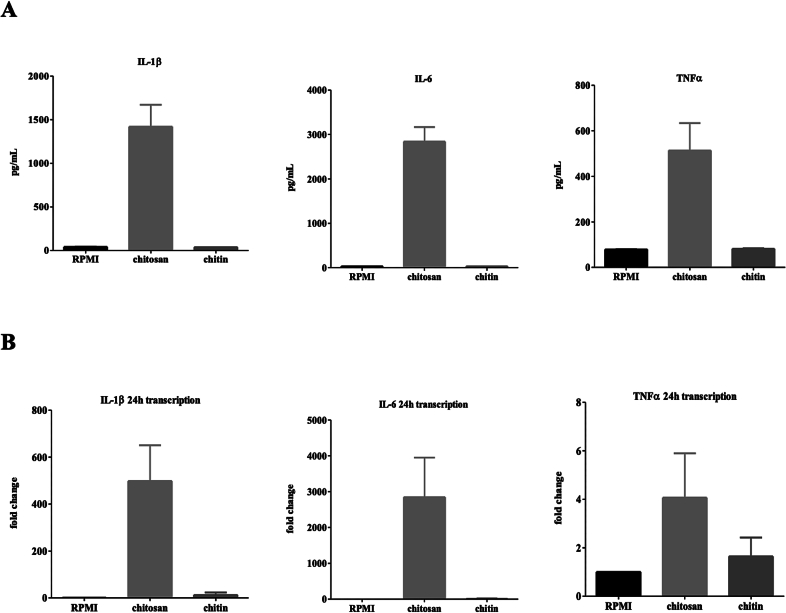
Chitosan induced pro-inflammatory cytokines in contrast to chitin. (A) IL-1β, IL-6, and TNFα production were measured in the cell culture supernatant of PBMCs of healthy controls that were stimulated with chitin (10 μg/ml) and chitosan (10 μg/ml) for 24 h without serum (*n* = 16). (B) IL-1β, IL-6, and TNFα transcription were measured by qPCR after 24 h (n = 16). Data shown are means ± SEM.

**Fig. 2:**
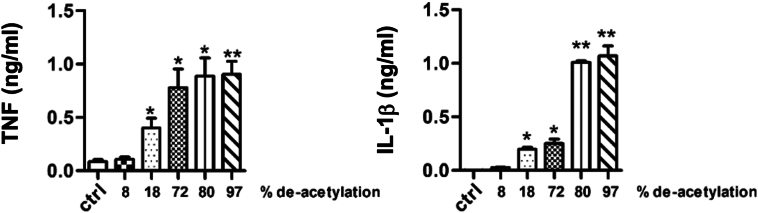
Pro-inflammatory cytokine induction depends on degree of deacetylation chitosan (also see Table 1 for origen of chitosan that correlates with deacetylation). PBMCs of healthy controls that were stimulated with chitosans with different levels of deacetylation(10 μg/ml) for 24 h without serum. IL-1β and TNFα production was measured with ELISA after 24 h. Data shown are means ± SEM.

First we analysed the immune response of hPBMCs to chitosan derived from chemically deacetylated *A. fumigatus* chitin. hPBMCs were stimulated with purified *A. fumigatus* chitosan or chitin for 24 h and the mRNA expression and the secretion of the pro-inflammatory cytokines IL-1β, IL-6 and TNF-α was analysed by qPCR and ELISA, respectively (Fig. 1). Transcription of the three analysed pro-inflammatory cytokines was upregulated 24 h after stimulation with chitosan, while lower transcription was induced by chitin (Fig. 1A). The chitosan-induced expression resulted in significant amounts of secreted IL-1β, TNF-α and IL-6 compared to the medium control or chitin-stimulated hPBMCs (Fig. 1B). Next, we wanted to determine the immunological properties of naturally occurring fl chitosans., with different degrees of deacetylation (DD) (Table 1) The secretion of IL-1β and TNF induced bychitosan in hPBMCs was analysed after 24 h of stimulation (Fig. 2). Chitosan with a low degree of deacetylation (DD 8 %) (close to chitin) did not induce a TNF or IL-1β cytokine response in hPBMCs, whereas an increasing induction of TNF and IL-1β secretion. (Fig. 2). Chitosans with the highest degree of deacetylation (DD 80–97 %) with elicited the highest secretion of the two analysed cytokines. Therefore, we conclude that chitosans, are a potent inducer of pro-inflammatory cytokines (e.g. IL-6, IL-1β, TNF) in hPBMCs and that the intensity of induction correlates with the degree of deacetylation of chitosan. (See Table 1.)

**Table 1:** Chitin and chitosan samples used.

Sample name	Source/Species	Strain	Degree of Deacetylation [DD] (%)	Reference/Source
*CrCH*	*Crustacea*	*Crab shell*	7.8 ± 2.8	Sigma-Aldrich
*CrCHO*	*Crustacea*	*Crab shell*	80 ± 5.0	InvivoGen
*A.f.*	*Aspergillus fumigatus*	1161	18.46 ± 1.57	1
*C.n.*	*Cryptococcus neoformans*	H99	72.44 ± 1.66	2
*M.c.*	*Mucor circinelloides*	CBS277.49	96.73 ± 0.77	3

### 3.2 CR3 is crucial for cytokine induction by chitosan

**Fig. 3:**
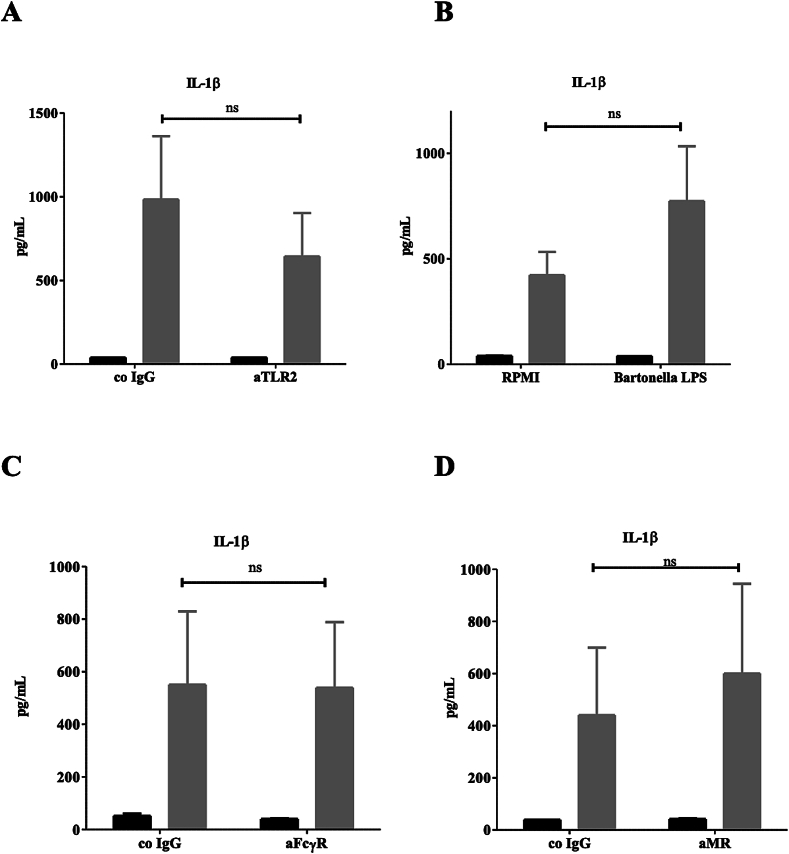
TLR2, TLR4, FcγR, and MR are not involved in chitosan induced responses. PBMCs of healthy volunteers were stimulated with RPMI or chitosan (10 μg/ml), after 1 h preincubation of a neutralizing antibody or molecule, (A) aTLR2 (10 μg/ml) for blocking TLR2, (B) Bartonella LPS (100 ng/ml) for blocking TLR4, (C) aFcγR (10 μg/ml) for blocking FcγRII, (D) aMR (10 μg/ml) for blocking mannose receptor. IL-1β was measured in the 24 h cell culture supernatant by ELISA. Data shown are means±SEM (*n* = 6). Statistical analysis was performed with the 2way ANOVA test.

**Fig. 4:**
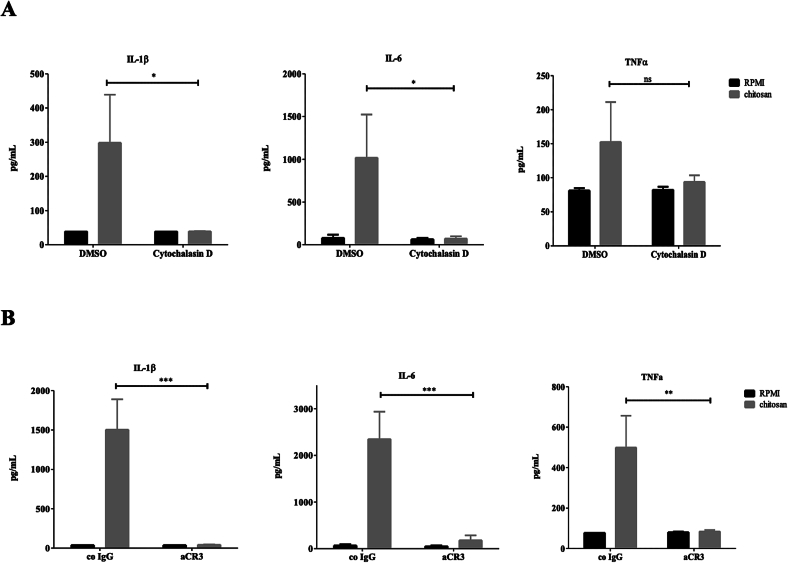
Chitosan induced pro-inflammatory responses are dependent on CR3 and phagocytosis. PBMCs of healthy volunteers were stimulated with RPMI or chitosan (10 μg/ml), after 1 h preincutation of (A) cytochalasin D (10 μg/ml) or DMSO, (B) isotype control or CR3 neutralizing antibody (10 μg/ml). IL-1β, IL-6, and TNFα production were measured in the cell culture supernatant after 24 h by ELISA. Data shown are means±SEM (*n* = 7). Statistical analysis was performed with the -way ANOVA test. * *p* < 0.05, **p < 0.01, ***p < 0.001.

**Fig. 5:**
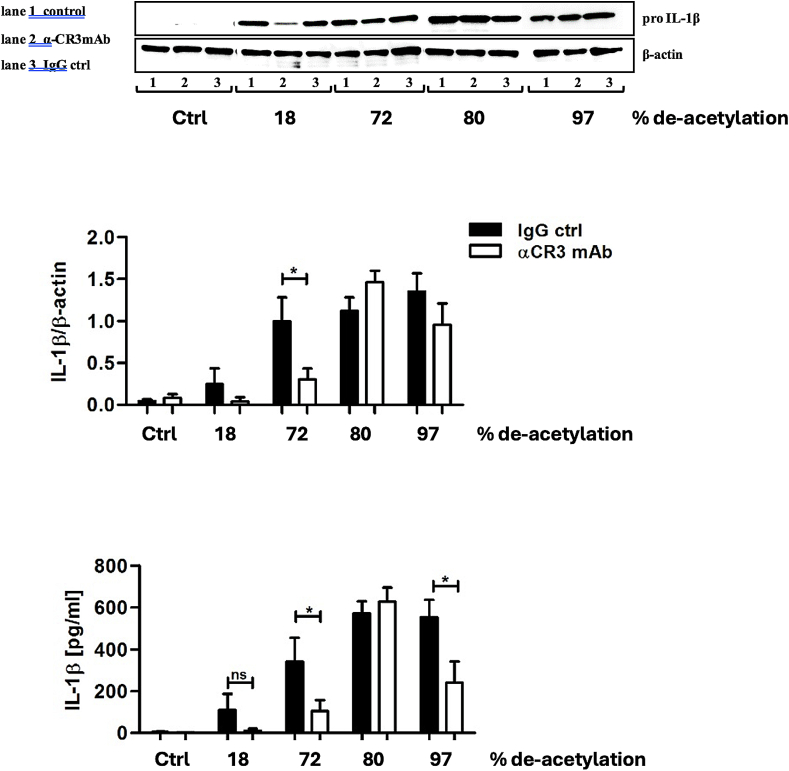
Western blotting for pro-IL-1b and production for IL-1b in the presence or absence of CR3 blocking after stimulation with chitosan with various degrees of deacetylation. (The origen and the corresponding degree of deacetylation is shown in Table 1).

Having identified pro-inflammatory cytokines induced by chitosan, we investigated the possible receptors that recognise chitosan. We first targeted receptors known to be involved in the recognition of fungal PAMPs, such as TLR2, TLR4, FcγR and the mannose receptor (MR) (Fig. 3). We stimulated hPBMCs with *A. fumigatus* chitosan and blocked either the PRRs TLR2 (Fig. 3A), TLR4 (Fig. 3B), FcγR (Fig. 3C), and MR (Fig. 3D). No difference in IL-1β induction was observed in the presence of the blocking antibodies or ligands indicating that these receptors are not involved in the recognition of chitosan. Next, we tested the hypothesis that the uptake of chitosan via phagocytosis is important for the observed IL-1β response. Blocking phagocytosis of PBMCs with the actin polymerisation inhibitor cytochalasin D (CytD) abolished IL-1β, IL-6 and TNF secretion by chitosan stimulated hPBMCs (Fig. 4A). This is in line with earlier studies (Bueter, Lee et al. 2011) and confirmed that chitosan must be phagocytosed to trigger downstream signaling. MR has been described to recognise chitin (Wagener, Malireddi et al. 2014) and since we did not observe any reduction in chitosan-induced IL-1β after blocking MR (Fig. 3D), we explored the possible role of another macrophage scavenger receptor; complement receptor 3 CR3. Blocking CR3 completely abolished all cytokine production induced by chitosan (Fig. 4B). CR3 blocked IL-1b induction induced by chitosan that was isolated from the cell wall of different fungi and crab shell and correlated with the degree of deacetylation (Fig. 5). Taken together, our findings indicate that phagocytosis and recognition via CR3 mediates pro-inflammatory cytokine production induced by chitosan.

### 3.3 Chitosan polarizes the aspergillus-Th response towards IL-22 production which is dependent on CR3

**Fig. 6:**
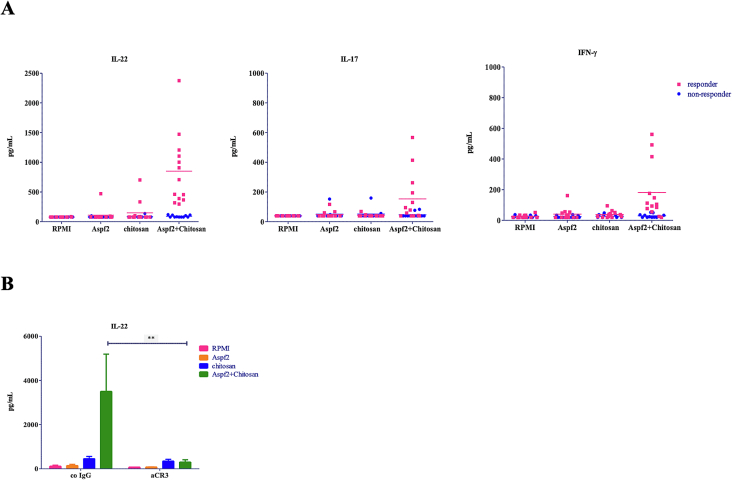
Chitosan synergizes with *Aspergillus* protein Aspf2, leading to high IL-22 production via CR3. (A) PBMCs of healthy volunteers were stimulated with chitosan (5 μg/ml), *Aspergillus* protein Aspf2 (1 μg/ml), and with the combination of them for 7 days in the absence human pooled serum. IL-22, IL-17 and IFNγ were measured in the cell culture supernatant by ELISA with 13 responders and 11 non-responders showing separately. (B) IL-22 was measured for the above conditions from the 13 responders, pretreated 1 h with CR3 neutralizing antibody (5 μg/ml) or its isotype control (5 μg/ml). Data shown are means ± SEM (*n* = 24). Statistical analysis was performed with the 2way ANOVA test.

To further explore the role of chitosan and its effects via CR3 in shaping antifungal host responses, we investigated whether adaptive immune responses could be modulated by chitosan, and whether this was dependent on CR3. Unlike *Aspergillus* conidia giving profound Th responses, chitosan itself did not trigger a Th response (Fig. 6A). Adaptive immune responses need a peptide presented by MHCII complex from antigen-presenting cell to naïve T cells that cooperate with the pro-inflammatory cytokines to shape the adaptive Th cells differentiation. Similar to a previously report where we investigated the capacity of *Candida* mannans to induce T helper responses with the use of the protein mp65 (van de Veerdonk, Marijnissen et al. 2009), we performed experiments with chitosan and the protein Aspf2 from *Aspergillus.* We stimulated PBMCs with chitosan in combination with Aspf2 for 7 days and measured Th17 cytokines IL-17, IL-22 and the Th1 cytokine IFNγ. Chitosan and Aspf2 alone did not induce a significant Th response, whereas in combination there was a clear induced T helper response (Fig. 6A), with especially the cytokine IL-22 being significantly induced. However, it was noted that only half of the healthy donors tested responded to the combination of chitosan and Aspf2. Next, we investigated whether similar receptors mediate the synergistic IL-22 production by the combination. We stimulated PBMCs from the responder donors with chitosan and Aspf2 after CR3 blocking and measured IL-22 after 7 days in the cell culture supernatant by ELISA. The synergy was completely blocked in the presence of CR3 antibody (Fig. 6B). Collectively, these data suggest that chitosan has the capacity to shape Aspf2-induced Th responses towards IL-22 production and this is dependent on CR3.

## 4 Discussion

Immunostimulatory capacities of chitin and its receptors have been investigated in several studies, showing receptors and pathways involving in its responses (Baker, Specht et al. 2011, Wagener, Malireddi et al. 2014, Wiesner, Specht et al. 2015, Upadhya, Baker et al. 2018, Lam, Upadhya et al. 2019). In contrast, only few studies investigated the immunological properties of chitosan, the deacetylated form of chitin,. Levitz et al. showed that chitosan leads to a robust IL-1β responses through a phagocytosis-dependent mechanism – but they did not identify a specific receptor for chitosan (Bueter, Lee et al. 2014). In the present study, we investigated the immunomodulatory responses of chitosan and its recognition by human immune cells. Chitin, by itself, did not induce any marked pro-inflammatory cytokine production in human PBMCs, neither in the absence or presence of human serum (Becker, Aimanianda et al. 2016). In contrast to chitin, chitosan induced significant proinflammatory cytokine production both with and without serum, independent of the activation of the complement system. This is in line with the finding that chitosan, but not chitin can activate the NLRP3 inflammasome, leading to robust IL-1β responses. Here we observed that blocking CR3 and phagocytosis significantly reduced chitosan-induced innate responses, which was independent of the activation of the complement system. CR3, also known as integrin α_M_β_2_ (Mac-1, CD11b/CD18), is expressed by monocytes and neutrophils. It recognises self-molecules such as complement but also pathogen-associated molecular patterns from pathogens such as β-1,3-glucan. Here we provide evidence that it thus can also interact with chitosan and modulate the antifungal host immune response.

IL-22, IL-17 and IFNγ are crucial for the host defence against human pathogens (Valeri and Raffatellu, 2016, Aggor et al., 2020, Lionakis, Drummond et al. 2023). We have previously shown that heat-killed *A. fumigatus* conidia induce Th1 and Th17 subsets in human PBMC, especially towatds a specific IL-22 producing Th subset (Gresnigt, Becker et al. 2013). Moreover, CR3 plays a significant role in the adaptive host defence against *Aspergillus*, although the ligands interacting with CR3 had not been identified. Based on our data, chitosan is likely to be a ligand for CR3 that subsequently modulates adaptive host responses against fungi, such as *Aspergillus*. Similar to our previous study investigating the capacity of *Candida* mannans to induce T helper responses using the protein Mp65 (ref), we conducted experiments with chitosan and selected the *Aspergillus fumigatus* protein Aspf2 due to its known immunogenicity and role of fungal virulence. Indeed, when chitosan alone was tested it did not induce Th responses, however, in combination with Aspf2, a protein from *A. fumigatus*, chitosan and Aspf2 synergized to induce Th1 and Th17 responses in the presence of human serum and this was dependent on CR3. This combination of a polysaccharide with a fungal derived protein from *Aspergillus* proved to be a potent inducer of IL-22 in cells from nearly half of the healthy volunteers. Next to our finding that CR3 might directly recognise and bind chitosan, our data on the Th subsets supports that CR3 also plays a role in Aspf2 triggered adaptive immune responses. This might be a more complex interplay since Aspf2 can recruit factors which in turn inactivates a very important ligand for CR3 namely C3b. The role of CR3 in shaping Th subsets is dependent on an active complement system in serum, Aspf2 and chitosan (Dasari et al., 2018). Aspf2 not only induces Th1 and Th17 responses via CR3, but also inhibits the activation of complement C3b, a key ligand of CR3. Therefore, future studies should investigate whether these seemingly contradictory phenomena represent different temporal states of the immune response.

This heralds the way for future work to investigate differences in the individual immune responses to *Aspergillus* exposure. Several diseases, such as fungal asthma or chronic pulmonary aspergillosis are predisposed in certain individuals and it would be interesting to explore and extrapolate this finding in a clinical context. Furthermore, in cells from responder donors, blocking CR3 resulted in complete inhibition of the synergy in IL-22 production, confirming CR3 involvement in the adaptive response to chitosan and making CR3 a potential target for modulating fungal host responses.

Unlike chitin, chitosan is always deacetylated from chitin, and is not a prevalent component in the cell wall of most fungi, although chitosan represents a significant fraction of the wall of *Cryptococcus* species and Zygomycetes (Lam, Upadhya et al. 2019, Gow and Lenardon, 2023). In *Cryptococcus* species the balance between chitin and chitosan plays a significant role in the immune response (Wiesner, Specht et al. 2015, Upadhya, Baker et al. 2018, Lam, Upadhya et al. 2019, Upadhya et al., 2021, Upadhya et al., 2023), and it is likely that chitosan is of significance to the immune responses during mucormycosis due to fungi of the genera *Mucor* and *Rhizopus*) (Cheng, Dickwella Widanage et al. 2024) and other fungi that deacetylate chitin during infection such as *Malassezia* species (Stalhberger et al., 2014). In addition, T cell function may also be affected by chitosan-fungal protein combinations and, for example, may modulate host responses in the lung triggered by *Aspergillus*. Collectively, it is reasonable to suggest that fungal chitosans elicit effects through CR3 to induce innate proinflammatory responses and that, chitosan, together with several *Aspergillus* protein such as Aspf2, might be a key trigger for adaptive *Aspergillus*-induced IL-22 responses through CR3. These data might have important implications for understanding the innate and adaptive immune responses to *Cryptococcus*, *Aspergillus, Rhizopus, Mucor, Malassezia* and other chitosan containing fungal pathogens.

Our study has several limitations. It is possible that chitosan size may influence the immune response as it does for chitin and β-1,3 glucan. Although we anticipated that here will be differing ranges of particle sizes in preparations, but did not seem to greatly influence the immune response. A comprehensive analysis of chitin, chitosan and β-1,3 glucan by purity and by fragment size is a major undertaking that still needs to be systematically addressed.

## CRediT authorship contribution statement

**Jeanette Wagener:** Conceptualization. **Xiaowen Wang:** Conceptualization. **Katharina L. Becker:** Conceptualization. **Vishu Aimanianda:** Conceptualization. **Isabel Valsecchi:** Conceptualization. **Mark S. Gresnigt:** Conceptualization. **Mihai G. Netea:** Conceptualization. **Jean-Paul Latge:** Conceptualization. **Neil A.R. Gow:** Conceptualization. **Frank L. van de Veerdonk:** Validation, Supervision, Data curation, Conceptualization.

## Declaration of competing interest

The authors declare that they have no known competing financial interests or personal relationships that could have appeared to influence the work reported in this paper.
